# Thirty-Third Annual Commencement of the Atlanta Medical College

**Published:** 1891-03

**Authors:** 


### THIRTY-THIRD ANNUAL COMMENCEMENT OF THE ATLANTA MEDICAL COLLEGE.

DeGive's Opera House was filled with a very large audience yesterday afternoon, when the closing exercises began. The faculty, in full force, occupied seats on the stage.

After a fervent prayer by Rev. W. D. Anderson, of the First Methodist church, Dr. W. S. Kendrick, proctor, then read the report of the faculty.

Following are the graduates :

W. E. Adams, J. J. Aderhold, N. Z. Anderson, J. T. Baggett, T. L. Ballenger, A. A. Barge, W. H. Bishop, W. A. Borders, F. W. Coile, J. W. Cook, C. O. Copelan, A. L. Cŭrtis, J. A. Farmer, P. H. Fitzhugh, W. B. Floyd, Clarence Freeman, E. J. Hesterly, A. S. Hill, C. Holmes, L. B. Bouchelle, Jr., J. T. Brice, R. E. Brown, H. S. Bruce, B. S. Burton, J. P. Carreker, A. F. Christopher, N. A. Frier, W. T. Grigg, A. P. Hanie, H. A. Hardeman, A. S. Harris, B. F. Hart, T. J. Hatchett, J. M. Head, W. T. Joiner, J. J. Bruce, G. O. Jones, J. W. Jordan, I. H. Lane, E. D. Hope, J. W. Howard, S. M. C. Howell, E. B. Hutcheson, J. H. Lattimore, A. A. Madden, G. W. Martin, W. J. Mathews, G. W. McCollum, J. K. McKinnon, E. F. Morrison, W. T. Newton, J. P. Norris, C. S. Northen, Frank Park, J. T. Pattillo, T. K. Quinn, N. T. Richardson, T. E. Rogers, W. P. Russell, F. V. Schley, R. B. Sconyers, H. R. Slack, R. E. Smith, K. A. Smith, S. A. Smith, J. F. Stewart, R. E. Stone, J. S. Sullivan, A. E. Vineyard, C. McK. Walker, J. A. Ward, J. J. Watkins, J. E. Watkins, J. A. Weaver, C. S. Webb, C. Q. West, G. M. Whitley, J. W. White, E. A. Winchester, J. A. Price, A. J. Didy.

Dr. J. A. Weaver, first honor; Dr. B. S. Burion, second honor; Dr. L. B. Bouchelle, Jr., third honor.

Honorable Mention.—Dr. J. F. Stewart, Dr. Frank Park, Dr. H. S. Bruce, Dr. R. E. Smith, Dr. F. W. Coille.

This report showed a most gratifying state of affairs, and showed that the Atlanta Medical College, one of the oldest and best medical colleges in the South, has made a splendid record during the past term.

To Hon. N. J. Hammond, the distinguished president of the board of trustees, befell the pleasant duty of conferring the degrees and delivering the diplomas.

Professor Charles H. Lane was introduced as orator, and proceeded to address the graduating class and the audience.

Dr. Frank Park followed with the valedictory, which was very beautifully rendered.

Rev. Dr. W. D. Anderson delivered the three handsome gold medals to those who won the banner, in a most appropriate address.

After the conclusion of the regular exercises there was a very pleasant little presentation episode.

Dr. H. R. Slack, of LaGrange, presented to Dr. Clarence Johnson, a handsome gold-headed cane, appropriately inscribed, as a small token of the ability and kindness of the recipient from the class of 1891.

Dr. Johnson replied in his usual happy style.

The exercises closed with prayer by Rev. Dr. W. D. Anderson.

---

